# Intubation Containment System for Improved Protection From Aerosolized Particles During Airway Management

**DOI:** 10.1109/JTEHM.2020.2993531

**Published:** 2020-05-11

**Authors:** Russell K. Gore, Christopher Saldana, David W. Wright, Adam M. Klein

**Affiliations:** 1 Department of Biomedical EngineeringGeorgia Institute of Technology 1372 AtlantaGA30332USA; 2 Shepherd Center 14586 AtlantaGA30309USA; 3 Department of Mechanical EngineeringGeorgia Institute of Technology 1372 AtlantaGA30332USA; 4 Department of Emergency MedicineEmory University 1371 AtlantaGA30322USA; 5 Department of OtolaryngologyEmory University 1371 AtlantaGA30322USA

**Keywords:** Personal protective equipment, biohazard containment, endotracheal intubation, resuscitation, aerosols

## Abstract

Objectives: Worldwide efforts to protect front line providers performing endotracheal intubation during the COVID-19 pandemic have led to innovative devices. Authors evaluated the aerosol containment effectiveness of a novel intubation aerosol containment system (IACS) compared with a recently promoted intubation box and no protective barrier. Methods: In a simulation center at the authors’ university, the IACS was compared to no protective barrier and an intubation box. Aerosolization was simulated using a commercial fog machine and leakage of aerosolize mist was visually assessed. Results: The IACS appeared to contain the aerosolized mist, while the intubation box allowed for mist to contact the laryngoscopist and contaminate the clinical space through arm port holes and the open caudal end. Both devices protected the laryngoscopist better than no protective barrier. Discussion: The IACS with integrated sleeves and plastic drape appears to offer superior protection for the laryngoscopist and assistant providers from aerosolized particles.

## Graphical abstract

Video of simulation trials 1-4 comparing no protection, the intubation box, and the intubation aerosol containment system (IACS). The IACS appears to contain the aerosolized mist, while the intubation box allowed for mist to contact the laryngoscopist and contaminate the clinical space through arm port holes and the open caudal end. Both devices protected the laryngoscopist better than no protective barrier.

## I. Introduction and Clinical Need

COVID-19 is primarily spread via small droplets generated during a cough or sneeze. However, certain procedures, such as intubation, increase the risk of aerosolizing large quantities of small and micro particles that are highly infectious and increase the risk to nearby providers. Worldwide efforts to develop personal protective equipment (PPE) for front line providers performing endotracheal intubation during the COVID-19 pandemic have led to innovative devices such as the intubation box [1], [2]. While this barrier has been shown to limit macroscopic contamination on the laryngoscopist, there is still concern for viral exposure through aerosolization of microscopic particles in several clinical settings. There is even greater concern for exposure in prehospital and emergency department settings where multiple providers may be simultaneously engaged in resuscitation efforts. This risk potentially threatens providers within 2 or more meters of the source and smaller aerosolized particles, with diameter less than $8\mu \text{m}$, may remain airborne for greater than 30 minutes [3]. During resuscitation procedures in these settings providers on the resuscitation team are in contact with or in close proximity to the patient for tasks such as chest compressions or assisting with airway management [4]. Thus, a solution protecting all providers on the resuscitation team from macroscopic contamination, as well as aerosolized microscopic viral particles, would be of benefit. This article introduces a novel protective barrier and assesses its ability to contain aerosolized mist compared with the intubation box and no protective barrier [1].

## II. Results

In 4 trials, the proposed IACS introduced in this article exhibited improved containment of the aerosolized mist. When compared with both no protective barrier and the intubation box, the IACS minimized contact with the laryngoscopist and no mist was observed escaping caudally into the clinical space.

In trial 1, with no protective barrier, mist can be seen spreading towards the laryngoscopist and into the surrounding clinical space during intubation of the simulation mannequin. In trial 2, with the intubation box, mist is observed escaping through the arm ports and the caudal end of the box. In trial 3, with the IACS, there is no mist observed escaping the IACS either toward the laryngoscopist or at the caudal end. In trial 4, a larger volume of mist was released to further test containment within the IACS. Again, no mist appears to escape the device.

## III. Methods

The proposed IACS is easily fabricated from a rigid polycarbonate barrier (PCB) with 2 circular arm ports and a thin, plastic drape attached to the superior and lateral edges of the barrier. Mounted on these circular arm ports are raised collars which enable attachment of flexible extension sleeves, currently stocked in most hospitals. The PCB has a ‘C’ shaped base (Fig. 2) allowing it to slide under the mattress of effectively any type of bed or stretcher, agnostic to bed size. As in the figure, the drape is attached to the boundary of the PCB and flows seamlessly over the patients torso and lower extremities, creating a protective barrier even during chest compressions. The upper portion of the PCB is tapered towards the patient allowing optimal airway landmark viewing during intubation.

**FIGURE 1.**
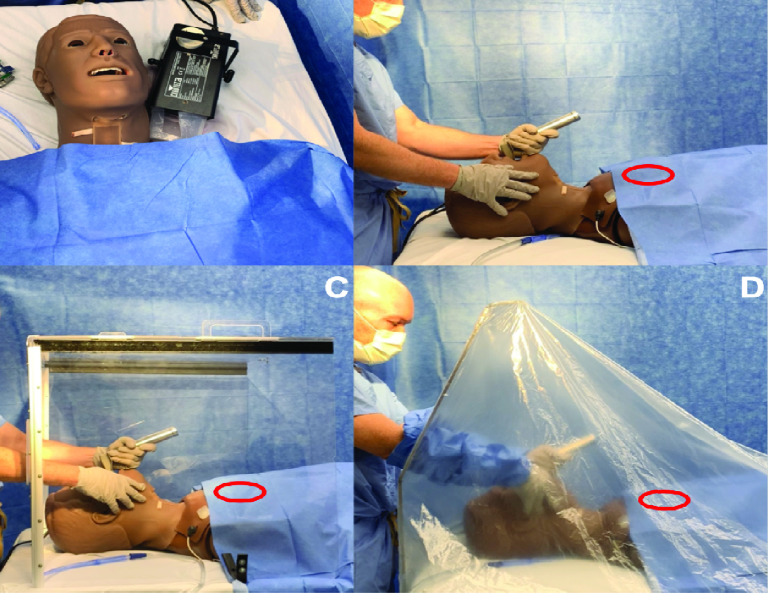
Experimental setup including position of equipment (A), position of laryngoscopist with no protection (B), intubation box (open arm holes) (C), and IACS (D). Anatomic landmark for chest compressions denoted with red oval. The pictured clinician provided permission for publication of image.

**FIGURE 2.**
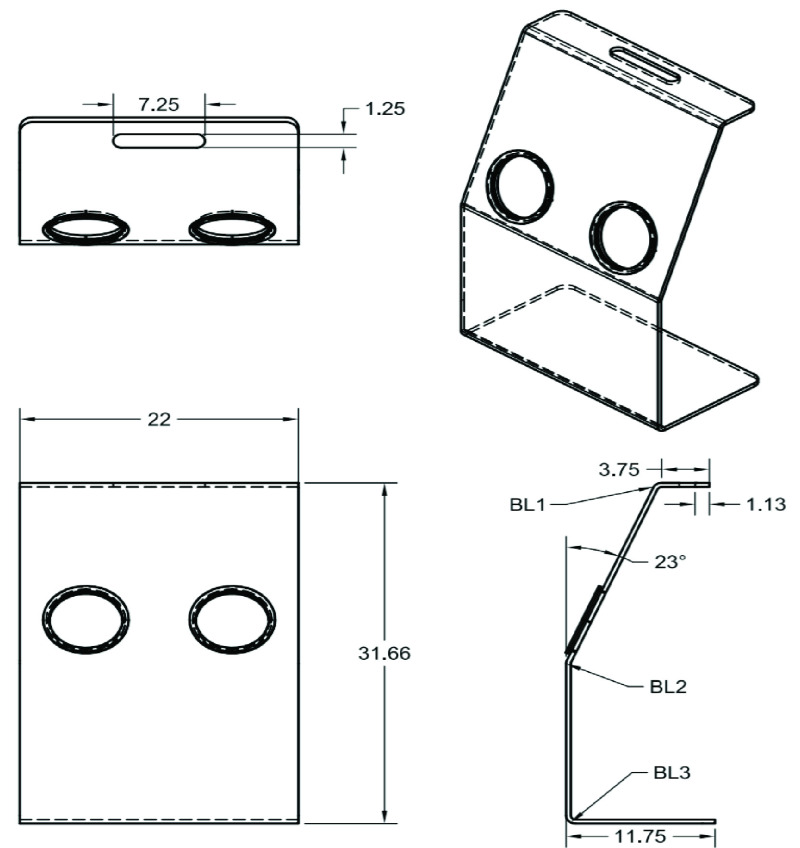
Intubation aerosol containment system (IACS) polycarbonate barrier (PCB) specifications and dimensions.

Trials were performed in a simulation center with a full size mannequin designed for simulating resuscitation. For each trial, a laryngoscopist stood at the head of the bed and reenacted a standard intubation on the mannequin. To simulate the spread of aerosolized particles a commercial fog machine (Hurricane 700, ChauvetDJ, Inc) was placed next to the head of the mannequin (Fig. 1). The fog generating mixture is a combination of water, triethylene glycol, and 1,2-propylene glycol (Bog Fog, Froggy’s Fog, Inc). Fog includes particles with diameters ranging from 0.1-$200\mu \text{m}$ [11]. A small container designed with a superior opening was placed over the outlet of the fog machine to direct the fog superiorly, thus simulating the vector of egress from the patient’s mouth. The container captured large droplets, resulting in condensation within the container, and the remaining mist exited through the superior opening. During each trial the fog machine was activated for 30 seconds. The experiment was performed serially with 1) no protective barrier, 2) an intubation box, and 3) the authors’ IACS (Fig. 1). In a single additional trial the capture container was removed during fog machine activation to further illustrate containment within the IACS (Video 2). Each trial was video recorded on an Apple iPhone XR with approximately 15 minutes between trials to allow fog to clear. Post processing of trial videos was performed on commercial video software (iMovie, Apple, Inc) and included cropping and applying a grey-scale color inversion filter to improve visualization of mist. Each trial video was synched to demonstrate similar periods of aerosolization over time (Video 1).

## IV. Discussion

In various healthcare settings (ambulance, ED, ICU, OR), patients may require resuscitation, intubation, or some form of upper airway management. The use of airway devices such as bag valve masks, laryngeal mask airways or endotracheal tubes result in aerosolization of airway fluids posing a significant transmission risk to providers and ancillary staff during these efforts [5]. Additionally, cardiopulmonary resuscitation (CPR) requires close contact with the patient to perform chest compressions, airway support and other life saving measures, depending on the circumstances. Such patients may have infections (such as COVID-19) either incidental to their current acute medical condition or as a result of such an infection, but typically, the infectious status of these patients is often unknown.

Risk from aerosolized microscopic viral particles is specifically relevant when managing patients with potential COVID-19 infection. Current evidence suggests that COVID-19 is associated with high viral loads in the upper respiratory tract and remains viable in aerosols for over an hour [6], [7]. Analysis of environmental samples in a biocontainment quarantine unit found evidence of diffuse environmental COVID-19 contamination including 66.7% of hallway air samples and 100% of in-room air samples [8]. Furthermore, COVID-19 is demonstrated to transmit even in asymptomatic carriers who may present for care due to other medical conditions or trauma [9]. The PPE donned by providers is critical, however, it may not provide adequate protection from large droplets or smaller aerosolized particles. Even with adequate PPE, emergency room providers were infected during the SARS coronavirus outbreak suggesting that masks alone do not prevent acquisition from environmental contamination [10]. Thus, there is a critical need for additional measures to protect healthcare providers during resuscitation and airway management. Such a solution must provide protection from aerosolized particles while allowing adequate exposure and contact with the patient to permit ongoing chest compressions, bag valve mask oxygenation, effective placement of an airway endotracheal tube, confirmation of tube placement and connection to a ventilator.

The IACS introduced in this correspondence is an inexpensive, reproducible protective barrier that appears to provide improved containment of aerosolized particles compared with no protection and the intubation box. The intubation box, now used in numerous countries around the world, is demonstrated to aid as a barrier to large droplets but results presented here suggest this design is not adequate to contain aerosolized particles [1]. In addition to present results suggesting improved protection from aerosolized particles, the IACS improves access to the patient for resuscitation team members. During resuscitation simulations, providers specifically noted limited patient access for resuscitation tasks when utilizing the intubation box. Using the IACS access was improved for tasks including providing airway assistance, suctioning, performing chest compressions and managing other injuries. Furthermore, the IACS is light weight, enabling quick deployment for use and rapid removal from the field in an emergent situation.

The methods employed in these simulations only approximated aerosolization of airway fluids with no control of droplet size a trajectory. Aerosolized particles move in complex patters based on multiple environmental factors such as room airflow and humidity which were not measured or controlled in these simulations. The presented simulations included a single trial for each experimental condition so results across multiple trials and variability in outcomes could not be assessed. Despite these limitations the results presented here suggest that the design of intubation containment systems should account for aerosolized contaminants in order to protect providers.

## V. Future Directions and Potential Clinical Impact

There is a critical need for innovative protective equipment for frontline providers. The proposed IACS may offer additional protection for healthcare providers during intubation and resuscitation, in multiple clinical settings, by decreasing environmental contamination from aerosolized particles and potentially decrease transmission of COVID-19 or other infectious airborne agents. It can be used during resuscitative efforts, intubation, extubation, or left in place during surgical procedures or in the ICU to reduce inadvertent spread from ventilator disconnections or endotracheal tube care. Future work will include further refinement and validation of the design to improve visualization of the patient and access by assisting providers. Further study is necessary, including prospective controlled clinical studies, to adequately assess the clinical significance of employing additional protective measures beyond standard PPE. Future efforts will explore safety considerations for IACS use. This includes assessing oxygenation and determining safe time limits within the IACS. Currently we recommend continuous clinical supervision within IACS and use only during resuscitation efforts or when mechanical ventilation is established. In addition, efforts will assess procedures for safely discarding potential contaminants within the IACS after resuscitation is complete to further minimize the likelihood of environmental contamination and infection transmission.

## Supplementary Material

10.21227/f0qs-ct30 INTUBATION AEROSOL CONTAINMENT SYSTEM VIDEO
